# Initial findings on RESTORE for healthcare workers: an internet-delivered intervention for COVID-19-related mental health symptoms

**DOI:** 10.1038/s41398-022-01965-3

**Published:** 2022-06-01

**Authors:** Kathryn Trottier, Candice M. Monson, Debra Kaysen, Anne C. Wagner, Rachel E. Liebman, Susan E. Abbey

**Affiliations:** 1 University Health Network, Toronto, ON Canada; 2 Department of Psychiatry, University of Toronto, Toronto, ON Canada; 3 Toronto Metropolitan University (formerly Ryerson University), Toronto, ON Canada; 4 Stanford University, Stanford, CA USA; 5 Remedy, Toronto, ON Canada

**Keywords:** Human behaviour, Psychiatric disorders

## Abstract

Many healthcare workers on the frontlines of the COVID-19 pandemic are experiencing clinical levels of mental health symptoms. Evidence-based interventions to address these symptoms are urgently needed. RESTORE (**R**ecovering from **E**xtreme **S**tressors **T**hrough **O**nline **R**esources and **E**-health) is an online guided transdiagnostic intervention including cognitive-behavioral interventions. It was specifically designed to improve symptoms of anxiety, depression, and posttraumatic stress disorder (PTSD) associated with COVID-19-related traumatic and extreme stressors. The aims of the present study were to assess the feasibility, acceptability, and initial efficacy of RESTORE in healthcare workers on the frontline of the COVID-19 pandemic. We conducted an initial uncontrolled trial of RESTORE in 21 healthcare workers who were exposed to COVID-19-related traumatic or extremely stressful experiences in the context of their work and who screened positive for clinical levels of anxiety, depression, and/or PTSD symptoms. RESTORE was found to be feasible and safe, and led to statistically significant and large effect size improvements in anxiety, depression, and PTSD symptoms over the course of the intervention through follow-up. RESTORE has the potential to become a widely disseminable evidence-based intervention to address mental health symptoms associated with mass traumas.

Clinical Trials Registration: This trial was registered with ClinicalTrials.gov ID: NCT04873622

## Introduction

There have been repeated calls for the development and evaluation of internet-delivered interventions to address mental health symptoms related to the COVID-19 pandemic (e.g., [1–3]). Effective interventions for healthcare workers (HCW) and others on the frontline of the pandemic who have been exposed to traumatic or extremely stressful experiences in the course of their work are urgently needed [4–8]. Given the large number of affected individuals, these interventions must be scalable to make a public health impact. In response to this need, our team developed a self-directed online intervention for individuals exposed to COVID-19-related traumatic and extreme stressors who are experiencing mental health symptoms [9]. RESTORE (**R**ecovering from **E**xtreme **S**tressors **T**hrough **O**nline **R**esources and **E**-health) is based on evidence-based cognitive-behavioral interventions designed to improve symptoms of anxiety, depression, and posttraumatic stress disorder (PTSD) related to COVID-19. The aims of the current study were to assess the feasibility, acceptability, and initial efficacy of RESTORE in healthcare workers on the frontline of the COVID-19 pandemic.

Research from the SARS and MERS outbreaks suggests that HCW who worked directly with infected patients were at risk for symptoms of anxiety, depression, and PTSD, particularly in the months and years that followed the outbreaks (e.g., [10]). A growing body of research indicates that globally, HCW on the frontlines of the COVID-19 pandemic are experiencing high rates of clinical levels of these mental health symptoms. Across Norway, Spain, France, the United Kingdom, and the United States, rates of at least moderate severity symptoms have been reported to be in the range of 24–70% for anxiety, 23–46% for depression, and 37–57% for PTSD [5, 11–15]. HCW who are nurses [15–18], are women, or of female sex [11, 16–18], and who worked directly with COVID-19 patients appear to be at highest risk (e.g., [12, 16, 17]).

These HCW have been exposed to higher risk of COVID-19 infection, have witnessed many deaths, and report emotional distress about restricting visitors to patients who were at the end of life or severely ill, factors that have been found to predict mental health symptoms in HCW during the COVID-19 pandemic [11, 19, 20]. RESTORE was specifically designed to address anxiety, depression, and PTSD symptoms related to exposure to these (and other) COVID-19-related extreme stressors. It was also specifically designed to engage HCW and others who may be reluctant to seek out mental health services [21] due to stigma, logistical challenges (e.g., scheduling), and other difficulties accessing treatment. This is accomplished through the self-directed nature of the intervention, use of non-pathologizing language, and guidance through direct messaging and/or calls.

Other internet-delivered mental health resources specific to COVID-19 have been developed and described in the literature (e.g., [22–28]). Many of these are educational and focus on building resilience and coping, and to date, no other intervention specifically targeting clinical mental health symptoms related to COVID-19 traumatic and/or extreme stressors has been reported. Very little data on outcomes of online COVID-19-related mental health interventions have been published. One exception is a randomized controlled trial (RCT) evaluating an unguided mobile phone app (PsyCovidApp) specifically designed to prevent and address anxiety, depression, stress, and burnout in HCW in the context of the pandemic [29]. PsyCovidApp is based on CBT and mindfulness approaches and consists of psychoeducational information, coping strategies, and suggested resources presented through written and audiovisual formats. The effects of two weeks of access to PsyCovidApp on anxiety, depression, and stress were compared to a control mental health app. PsyCovidApp showed small but statistically significant benefits over the control app for individuals receiving psychotherapy or taking psychotropic medication. To our knowledge, the present paper is the first to report outcomes of a guided internet-delivered intervention specifically designed to address clinical levels of mental health symptoms associated with exposure to COVID-19-related traumatic and/or extreme stressors.

This study reports on an uncontrolled trial of RESTORE in HCW who were exposed to COVID-19-related traumatic or extremely stressful experiences and who endorsed moderate to severe symptoms of anxiety, and/or depression, and/or PTSD symptoms. We anticipated that RESTORE would be feasible (i.e., that recruitment would be successful, and that there would be good adherence and engagement with the intervention), acceptable to participants (i.e., that participants would be satisfied with the intervention), and efficacious in decreasing symptoms of anxiety, depression, and PTSD.

## Method

### Design

The study was an uncontrolled trial with participants assessed at baseline, mid-intervention (i.e., after module 4), end-of-intervention (i.e., after module 8), and at 1-month follow-up. An intent-to-treat approach, hereafter referred to as intent-to-intervene (ITI) given the self-directed nature of the intervention, to data collection was utilized. Participants who did not complete the intervention were still asked to complete the remaining assessments and all available data were included in the analyses.

### Participants

Twenty-two participants were recruited and consented to participate between March 20th and May 25th, 2021. Participants were recruited through email advertisement at University Health Network in Toronto, ON, Canada and through outreach to other relevant organizations (e.g., presentations to long-term care centers, advertisements in newsletters to HCW and first responders). All participants resided in Ontario, Canada. One participant did not complete the baseline assessment and therefore was not enrolled in the intervention. As a result, they were excluded from the analyses, leaving a sample size of *N* = 21. Inclusion criteria were: being a Canadian HCW, first responder, or military member who experienced a traumatic or extremely stressful event related to COVID-19 in the course of their work, as well as moderate or more severe symptoms of anxiety, and/or depression, and/or PTSD symptoms assessed via standardized self-report questionnaires (see the Participants section below for details). All individuals who signed-up to be considered for the study were HCWs. Participants also needed to be fluent in English. Exclusion criteria were: elevated risk of suicide defined by a suicide attempt in the past year or endorsement of more than brief thoughts of suicide in the past week, lack of access to high-speed internet, and current participation in psychotherapy or another intervention targeting stress responses related to COVID-19.

### Procedure

**Fig. 1:**
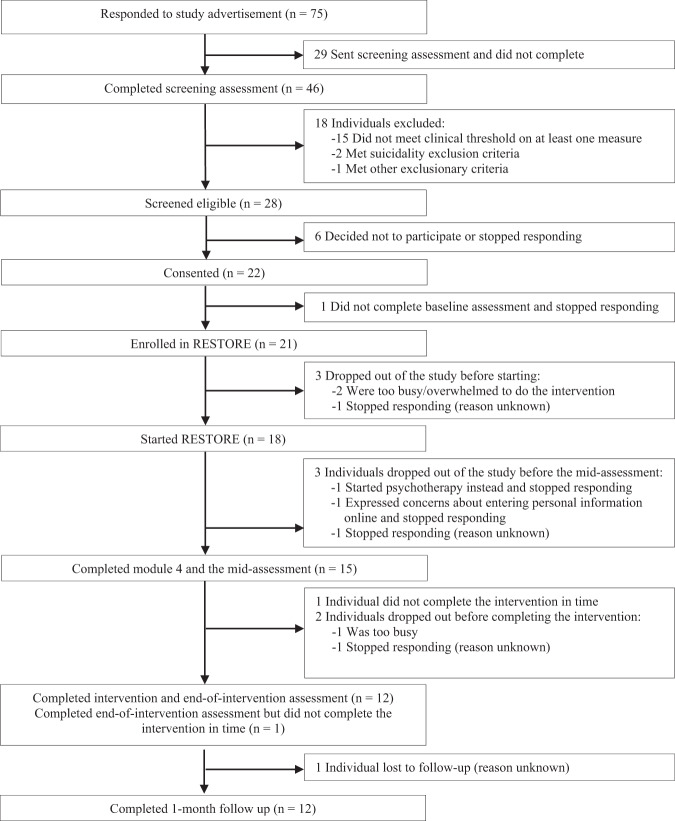
Flow diagram of participants through the phases of study recruitment, assessment, and intervention.

Interested individuals signed up on the RESTORE website and were then sent an electronic link to the study screening consent information and measures. Individuals who met the eligibility criteria then underwent the informed consent process via a telephone call with the study coordinator. Those who consented were subsequently sent an electronic link to the baseline assessment measures. Following completion of these measures, participants were enrolled in RESTORE and provided with their confidential log-in information. All assessments were administered electronically via REDCap. Recruitment coincided with the peak of the third and most significant wave (to date) of COVID-19 in Ontario, Canada. During the time of this study, HCWs on the frontlines of the pandemic were experiencing high workloads, frequent deaths related to COVID-19, policies restricting visitors to patients, and redeployment to different work environments and tasks. Participant flow is depicted in Fig. 1.

### Intervention

RESTORE is an online, guided, self-directed mental health intervention based on evidence-based cognitive-behavioral therapies (CBT) for individuals exposed to traumatic or other extremely stressful experiences. It is unique in that it is transdiagnostic and was specifically developed for the COVID-19 context. An overview of the content of the 8 RESTORE modules and further detail regarding intervention development are described by Trottier et al. [9]. In short, RESTORE is designed to address three main potential mechanisms that we hypothesized may cause and maintain mental health symptoms in those exposed to COVID-19-related traumatic or extreme stressors: (1) social isolation and withdrawal from other positive activities, (2) avoidance of thoughts, feelings, and situations related to extremely stressful or traumatic events, and (3) negative cognitions about the cause, meaning, and implications of traumatic or extremely stressful events. We primarily adapted Cognitive Processing Therapy [30], an evidence-based psychotherapy for PTSD, to facilitate acceptance of the extremely stressful events related to COVID-19, and to shift negative beliefs about the implications of the events. We also included graded exposure [31] and positive activity scheduling [32], two well-established evidence-based psychotherapy interventions for anxiety and depression. After working through each module, users are given practice assignments to work on before moving on to the next module. Each module starts with users completing self-report measures of anxiety, depression, and PTSD and receiving feedback on their scores. The modules and practice assignments consist of a combination of written information, brief informational videos, interactive examples and exercises, self-monitoring (e.g., scheduling and recording positive activities), and questions for users to reflect on and respond to using free text boxes (e.g., reflecting and writing about how the worst events of the pandemic affected thoughts, feelings and behaviors). Every user receives the same intervention components. Participants were given up to 8 weeks to complete the intervention but were encouraged to complete the modules at a pace of two per week given recent research suggesting that more frequent CBT sessions for PTSD predicts better outcomes [33].

RESTORE includes guidance by a non-psychotherapist in order to increase engagement and completion while also increasing scalability of the intervention [34] and to overcome access issues related to psychotherapist availability and jurisdictional issues related to licensure/registration. The guide’s role primarily involves reviewing symptom change with the participant, reinforcing practice assignment completion and improvements, enhancing motivation for engagement, and troubleshooting barriers to improvement, module completion, and/or engagement with the program (e.g., practice assignment avoidance). Guides encourage engagement and module completion by praising participants’ work done on the modules, reaching out through direct messaging to encourage re-engagement if progress stalled, and by instilling hope that RESTORE may help to improve stress reactions (e.g., “I really want you to have the opportunity to benefit from the program.”). If participants are not on track to complete a module in time for their scheduled check-in, the guide reaches out to encourage completion. The guide and user can choose to reschedule a check-in to within a few days time to allow time for completion. If a participant still does not finish the module, the guide moves forward with the check-in and enhancing engagement and problem solving module completion are addressed in the check-in. In the current study, the three study guides had at least a bachelor’s degree and a background in psychology, and were supervised by the first and fourth authors. Guides were trained in two 2-hour workshops on the guidance manual, spent ~4 h reviewing the online platform and practicing guidance calls before, and in between, the workshops, and attended weekly group supervision sessions. Participants received 5 brief check-ins with their guide either via direct messaging or a call (according to the participant’s preference) after modules 1, 2, 4, 6, and 8. One “as-needed” call was also available at any time during the program should it be requested by a participant or deemed necessary by their coach (e.g., participant has not engaged with the platform for 2 weeks). In the current study, no participants received an as-needed call. Guides were also accessible via secure messaging on the RESTORE platform.

### Measures

#### Mental health assessment

Given the transdiagnostic nature of the intervention, the primary outcomes for the study were self-reported anxiety, depression, and PTSD symptoms over the past week. The following reliable and well-validated measures were used to assess eligibility and were the primary outcome measures for the study: the Generalized Anxiety Disorder-7 (GAD-7; [35]) was used to assess symptoms of generalized anxiety (range 0–21; eligibility threshold ≥ 10; *α*s = 0.87−0.90); the Patient Health Questionnaire-9 (PHQ-9; [36]) was used to measure depressive symptoms (range 0–27; eligibility threshold ≥ 10; *α*s = 0.86–0.90), and the Posttraumatic Stress Disorder Checklist-5 (PCL-5; [37]) was used to assess PTSD symptom severity (range 0–80; eligibility threshold ≥ 33; *α*s = 0.87–0.95). In order to be eligible, individuals needed to score above the clinical threshold on at least one of these measures. On all three measures, higher scores reflect higher symptom severity. Past week versions of all measures were used in the current study.

Suicide risk was assessed through a two-item screener which was adapted from the National Institute of Mental Health’s Ask Suicide-Screening Questions [38] and from the Beck Scale for Suicidal Ideation [39]. Individuals were considered to be at elevated risk and excluded if they endorsed more than “brief” thoughts of suicide in the past week or if they reported a suicide attempt in the past year.

#### Feasibility and acceptability

Feasibility of recruitment, adherence to the intervention, intervention engagement, and participant satisfaction were defined a priori as indicators of feasibility and acceptability. Feasibility of recruitment was measured by percentage of screened individuals who were deemed eligible and percentage of those who screened eligible who were subsequently enrolled. Adherence to the intervention was measured by mean number of completed modules and drop-out rates. Intervention engagement was measured by mean number of RESTORE log-ins and text entries into the platform (e.g., typed responses to questions, self-monitoring entries). The first 6 items from the Client Satisfaction Questionnaire-8 (CSQ-8 [40]; α = 0.94) were used to evaluate acceptance (i.e., satisfaction) with the RESTORE intervention (Items 7 and 8 were omitted from the CSQ-8 due to an administrative error). Scores could range from 6 to 24 with higher scores reflecting higher client satisfaction.

### Statistical analyses

We conducted analyses of outcomes on the intent-to-intervene and intervention completer samples (i.e., those who completed all eight modules of the intervention; *n* = 12). Changes in the three primary outcomes of interest were tested in SPSS version 28 [41] using multilevel growth models estimated with restricted maximum likelihood. A Kenward–Roger correction for small samples was used to correct bias in regression coefficients and standard errors [42]. Multilevel models account for dependency in repeated measures and have the ability to use all available data to account for missing data. Each outcome was regressed onto time coded as assessment interval (i.e., baseline, mid-intervention, end-of-intervention, and 1-month follow up). Random intercepts and slopes were included in all models. Standardized effect size estimates represent model-estimated change from baseline to end-of-intervention and 1-month follow up, divided by the baseline standard deviation of the outcome variable [43], with a *Hedges g* correction for small samples [44].

## Results

### Participant characteristics, feasibility, and acceptability

As shown in Table 1, the majority of the participants were nurses and identified as women. Mean age of the sample was 39.10 (*SD* = 11.48, range = 22–59). Mean hours worked per week was 44.74 (*SD* = 11.78, range = 24–75). Income and ethnicity varied across the sample. There were no participants withdrawn for safety-related reasons. Eighty-one percent of participants screened positive on the GAD-7, 90.5% screened positive on the PHQ-9, and 95.2% on the PCL-5; 76.2% of participants screened positive on all three measures. Approximately, 55% of participants chose to have guidance check-ins through direct messaging, 28% chose guidance check-ins through calls, and 17% had check-ins through a combination of direct messaging and calls. There were no suicide attempts or psychiatric hospitalizations reported by the participants over the course of participation in the intervention. On average, the ITI sample completed 5.33 modules (*SD* = 3.45). Twelve individuals completed all 8 modules, 3 completed four or five modules, and 2 completed one module. On average, the ITI sample logged into RESTORE 22.95 (*SD* = 15.85) times and made 56.67 (*SD* = 39.72) text entries in the modules. The intervention completer sample logged in a mean of 32.17 (*SD* = 9.69) times, and made 84.42 (*SD* = 20.36) entries. Mean client satisfaction with the program was 20.7 out of 24 (*SD* = 3.80) in the ITI sample and 20.5 out of 24 (*SD* = 3.91) in the completer sample.

**Table 1:** Sample demographic and mental health characteristics.

	*N*	*%*
Gender		
Woman	20	95.2
Man	1	4.8
Ethnicity		
Black	1	4.8
East/Southeast Asian	4	19.0
South Asian	2	9.5
White	6	28.6
Another race category	1	4.8
Declined to provide	7	33.3
Work setting		
Hospital	16	76.2
Long-term care	2	9.5
Declined to provide	3	14.3
Current occupation		
Administrative	4	19.0
Nursing	11	52.4
Personal support	3	14.3
Respiratory therapist	2	9.5
Security	1	4.8
Education		
Some college/university	1	4.8
College diploma	6	28.6
Undergraduate degree	8	38.1
Master’s degree	5	23.8
Declined to provide	1	4.8
Income		
$35,000–$49,999	2	9.5
$50,000–$74,000	6	28.6
$75,000–$99,000	3	14.3
$100,000–$249,999	7	33.3
$250,000+	3	14.3
Marital status		
Single	8	38.1
Married/Cohabitating	12	57.1
Separated	1	4.8
Lifetime mental health diagnosis		
Anxiety	6	28.6
Depression	4	19.0
PTSD	1	4.8
Lifetime psychotherapy	8	38.1
Psychotropic medication	7	33.3
Guidance check-in format chosen		
Direct messages	10	47.6
Audio calls	5	23.8
Both	3	14.3

### Mental health outcomes

Table 2 shows descriptive statistics of the primary outcomes at each time point and effect size estimates of change in the primary outcomes from baseline to end-of-intervention and baseline to 1-month follow up for the ITI and intervention completer samples, respectively. In both samples, there were significant improvements in participants’ self-reported past week anxiety (ITI: *B* = −3.07, *SE* = 0.46, *p* < 0.001, baseline to follow-up *g* = 1.58; completer: *B* = −3.13, *SE* = 0.55, *p* < 0.001, baseline to follow-up *g* = 1.26), depression (ITI: *B* = −3.00, *SE* = 0.52, *p* < 0.001, baseline to follow-up *g* = 1.34; completer: *B* = −3.19, *SE* = 0.59, *p* < 0.001, baseline to follow-up *g* = 1.29) and PTSD severity (ITI: *B* = −9.70, *SE* = 1.13, *p* < 0.001, baseline to follow-up *g* = 1.85; completer: *B* = −9.63, *SE* = 1.31, *p* < 0.001, baseline to follow-up *g* = 1.63).

**Table 2:** Estimated marginal means from multilevel models, raw standard deviations, and Hedges *g* effect sizes from baseline to end-of-intervention and 1-month follow up for intent-to-intervene, intervention starter and intervention completer samples.

	Baseline *M (SD)*^a^	Mid *M (SD)*	EoI *M (SD)*	FU *M (SD)*	Baseline to EoI Hedges *g*^b^	Baseline to FU Hedges *g*^b^
Intent-to-intervene sample (*n* = 21)						
Anxiety	11.12 (5.32)	8.06 (4.93)	4.99 (3.59)	1.93 (4.05)	1.05	1.58
Depression	12.53 (6.15)	9.54 (5.54)	6.54 (4.75)	3.54 (4.54)	0.89	1.34
PTSD	34.17 (14.39)	24.48 (11.44)	14.78 (10.44)	5.08 (10.19)	1.23	1.85
Intervention completer sample (*n* = 12)						
Anxiety	11.12 (6.27)	7.98 (5.09)	4.85 (3.70)	1.72 (4.23)	0.84	1.26
Depression	13.13 (6.25)	9.94 (5.88)	6.75 (4.81)	3.55 (4.69)	0.86	1.29
PTSD	34.16 (14.93)	24.53 (12.23)	14.89 (10.59)	5.26 (10.32)	1.09	1.63

*EoI* end of intervention, *FU* follow up, *PTSD* posttraumatic stress disorder.

^a^Estimated marginal means from multilevel models and raw standard deviations.

^b^Effect sizes represent estimated change from the multilevel model in outcome variable from baseline to end-of-intervention and baseline to 1-month follow-up divided by the raw standard deviation in the outcome variable at baseline with a correction for small sample sizes [44].

## Discussion

In this initial trial, we found that recruitment, delivery, and evaluation of RESTORE in HCWs on the frontline of the COVID-19 pandemic was feasible and safe. HCW in the current study were characterized by high levels of anxiety, depression, and PTSD symptoms with most participants having elevated symptoms in all three domains. Improvements in anxiety, depression, and PTSD symptoms over the course of the intervention were of large magnitude. Moreover, examination of the effect sizes indicated further improvement over the 1-month follow-up period consistent with RESTORE’s focus on teaching skills and encouraging practice both over the course of the intervention and afterward. Effect sizes were large in both the ITI sample and completer samples, suggesting that even those who did not complete the full intervention benefited. That said, although those who did not complete the intervention were still asked to do the remaining assessments, most did not, which may have contributed to larger effect sizes in the ITI sample. This raises the question of how much of the intervention is needed to lead to good outcomes, and if those who didn’t complete RESTORE may have stopped because they had gotten what they needed. Future research will examine patterns of response in those who do not complete.

Overall, the findings from this initial trial are promising in that the magnitude of the benefits from this online self-directed intervention are in line with those found with individual CBTs [45–47], which require trained therapists and can be difficult and expensive to access. Moreover, the benefits were found across three mental health outcomes. As a transdiagnostic intervention, RESTORE may be effective across a range of mental health symptoms following exposure to COVID-19-related traumatic and other extreme stressors.

It is important that the observed outcomes of RESTORE in this study be considered within the context of the timing of delivery. The timing of the study coinciding with a surge in COVID-19 cases and hospitalizations was not intentional. On the whole, participants described experiencing repeated and ongoing COVID-19 related traumatic and extreme stressors in the course of their work. We had concerns about the ongoing exposure to these stressors negatively affecting outcomes due to the impact of not being “post-exposure”. At the same time, other studies have found that trauma-related symptoms of anxiety, depression, and PTSD can be treated even in situations with ongoing exposure to stressors (see ref. [48] for review). Given that the ongoing COVID-19 pandemic is characterized by periods of increased surges in cases and corresponding exposure to both general and extreme stressors for healthcare workers, our finding that RESTORE can improve symptoms of anxiety, depression, and PTSD in the midst of a surge in cases bodes well for this and other interventions.

We also thought that we may see low adherence and engagement with the intervention due to the impact of the surge in COVID-19 cases and hospitalizations. Three participants who enrolled dropped out before ever starting the intervention. Two of these individuals cited not having the time and energy to devote to the program due to the surge in cases as the reason for not starting (the other didn’t provide a reason), and a number of participants reported feeling overwhelmed and exhausted by their work over the course of the intervention. The rates of not starting (14.3%) and non-completion after starting (29.4%) were moderate and comparable to other guided online interventions and individual CBTs for anxiety, depression, and PTSD [49–51]. Taken together, it appears that having conducted the study during a surge in COVID-19 cases and hospitalizations did not negatively affect intervention engagement or outcomes.

The uncontrolled design of the current study is a significant limitation. An alternative explanation for the improvements seen over the course of using the intervention, and the further improvements seen over follow-up, is that participants may have been on a natural course of recovery and they may have improved regardless of having participated in RESTORE. Indeed, most individuals recover naturally following traumatic events with only a minority going on to have clinically significant mental health symptoms months and years later [52, 53]. A RCT is needed to rule out this alternative explanation and this is our next step in testing RESTORE in this population.

In sum, findings from this initial uncontrolled trial suggest that RESTORE is safe, feasible, and acceptable, and may be efficacious at improving symptoms of anxiety, depression, and PTSD associated with COVID-19-related traumatic and other extremely stressful experiences in frontline HCWs. To our knowledge, these are the first published outcome data from an intervention of this nature in the context of the COVID-19 pandemic. This is important given the extent of the need and the repeated calls for an intervention that can address the burden the COVID-19 pandemic has caused on individuals, the healthcare system and society. We believe that RESTORE has the potential to become a widely available and evidence-based intervention to address negative mental health effects of the COVID-19 pandemic.
